# The role of 2D/3D spin-polarization interactions in hybrid copper hydroxide acetate: new insights from first-principles molecular dynamics

**DOI:** 10.3762/bjnano.8.86

**Published:** 2017-04-12

**Authors:** Ziyad Chaker, Guido Ori, Mauro Boero, Carlo Massobrio

**Affiliations:** 1 Université de Strasbourg, CNRS, Institut de Physique et Chimie des Matériaux de Strasbourg, UMR 7504, F-67034 Strasbourg, France

**Keywords:** first-principles molecular dynamics, hybrid material, magnetic properties, pressure, spin polarization

## Abstract

The magnetic properties response of the layered hybrid material copper hydroxide acetate Cu_2_(OH)_3_CH_3_COO·H_2_O is studied as a function of the applied pressure within first-principles molecular dynamics. We are able to elucidate the interplay between the structural properties of this material and its magnetic character, both at the local (atomic) level and at the bulk level. We performed a detailed analysis of the intralayer spin configurations occurring for each value of the imposed projection along the *z*-axis for the total spin and of the applied pressure. The transition from an antiferromagnetic to a ferromagnetic state at high pressure (above 3 GPa) goes along with a vanishing difference between the spin polarizations pertaining to each layer. Therefore, at high pressure, copper hydroxide acetate is a ferromagnet with no changes of spin polarization in the direction perpendicular to the inorganic layers.

## Introduction

Copper hydroxide acetate Cu_2_(OH)_3_CH_3_COO·H_2_O (CuOHAc) is the precursor of a whole class of hybrid organic–inorganic materials that are made of inorganic sheets separated by alkyl chains (such as alkyl sulfates and carboxylates) or conjugated molecules (such as fluorene phosphonates) [1–3]. The interest in Cu_2_(OH)_3_X systems stems from their tunable magnetic properties, strongly dependent on the nature of the organic ligands. Based on experimental evidence, CuOHAc exhibits (when seen as a bulk material) 3D antiferromagnetic (AF) interlayer character and (within each layer) a weak ferromagnetic (F) intralayer (2D) character. The use of pressure is a valuable and practical tool to tune the magnetic behavior of this lamellar hybrid material [4]. In our previous studies, first-principles molecular dynamics (FPMD) approaches combined with density functional theory (DFT) have been useful to complement the missing structural information on the atomic positions obtained experimentally [5]. We also provided an exploratory insight into CuOHAc bonding and magnetic properties changes in response to an applied external pressure [6–7]. Recently, Banerjee et al. [8–9] with a similar approach by means of first-principles calculations showed a cooperative spin-state transition upon application of pressure for a hybrid metal–organic framework perovskite. Both the transition pressure and the width of the hysteresis have found to be strongly dependent on the nature of the organic molecular component.

Motivated by an updated set of the atomic coordinates for CuOHAc, recently proposed on the basis of a single-crystal X-ray experiment [10], we refine here the study of the effect of pressure on the structural and 2D/3D magnetic character of CuOHAc. With respect to previous theoretical studies, we consider a supercell four times larger and a wider range of pressures (0–17 GPa) as well as magnetization states for three different values of the spin multiplicities. Overall, this novel set of calculations allowed us to achieve a better agreement with the experimental findings in terms of the AF-to-F transition pressure. We also elucidate in greater detail the role of spin polarization in driving the AF-to-F transition, thereby making available an atomic-scale rationale for the change of magnetic nature.

## Computational Methods

We employ a first-principles molecular dynamics (FPMD) approach in the framework of Kohn–Sham density functional theory (DFT) with a generalized gradient approximation according to Becke [11] for the exchange energy and according to Lee, Yang and Parr [12] (BLYP) for the correlation functional. The valence electrons are treated explicitly, whereas norm-conserving pseudo-potentials generated following the scheme of Trouiller and Martin [13] are used to account for the core–valence interaction. Nonlinear core corrections (NLCC) are used for Cu atoms according to the prescription of Louie and co-workers [14]. The present calculations are performed on a system made of 288 atoms (32 Cu, 96 O, 32 C, and 128 H) applying periodic boundary conditions. Such a supercell is the result of doubling the primitive cell of 72 atoms along the *x*- and *y*-directions. The original lattice parameters *a* = 5.5776 Å, *b* = 6.0733 Å, *c* = 18.5134 Å and β = 91.802°, and symmetry group *P*2_1_ are those recently proposed by Svarcova and co-workers [10] based on a single-crystal X-ray diffraction study. The wave functions are expanded at the Γ point of the supercell in a plane-wave basis set with an energy cutoff of 90 Ry. Following our previous works [6–7], in order to produce a set of distinct electronic ground states differing in the local spin densities, we assigned different initial random values to the fictitious electronic degree of freedom. In particular, we performed 17 initial randomization per pressure and per magnetization state. More details about the procedure employed can be found in [5]. This procedure allows us to explore the multiple-minima landscape resulting from the existence of different spin configurations for a given value of the imposed multiplicity. Each multiplicity corresponds to a total value *S* for the projection along the *z*-direction of the total spin. In this way one obtains different local intralayer spin configurations and associated values Σ (Σ = 0 or Σ ≠ 0) corresponding to an identical multiplicity for the whole system. Herein, we consider both limiting cases of total spin multiplicity 2*S* + 1 corresponding to values equal to 1 (*S* = 0) and 33 (*S* = 16) as well as one intermediate case such as 17 (*S* = 8). The spin density is defined as


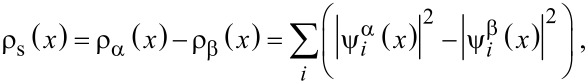


where α and β indicate, respectively, the up- and down-spin components, and 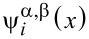 are the Kohn–Sham orbitals of the system. The systems under pressure are obtained by reducing the dimensions of the model on the three crystallographic directions. This has been done by following the experimental changes of the lattice parameters reported in [4]. For each system the pressure is then computed by fitting the total energy *E* of the system as a function of the volume *V* of the simulation cell following the definition *P*_ext_ = d*E*/d*V*.

## Results and Discussion

**Figure 1:**
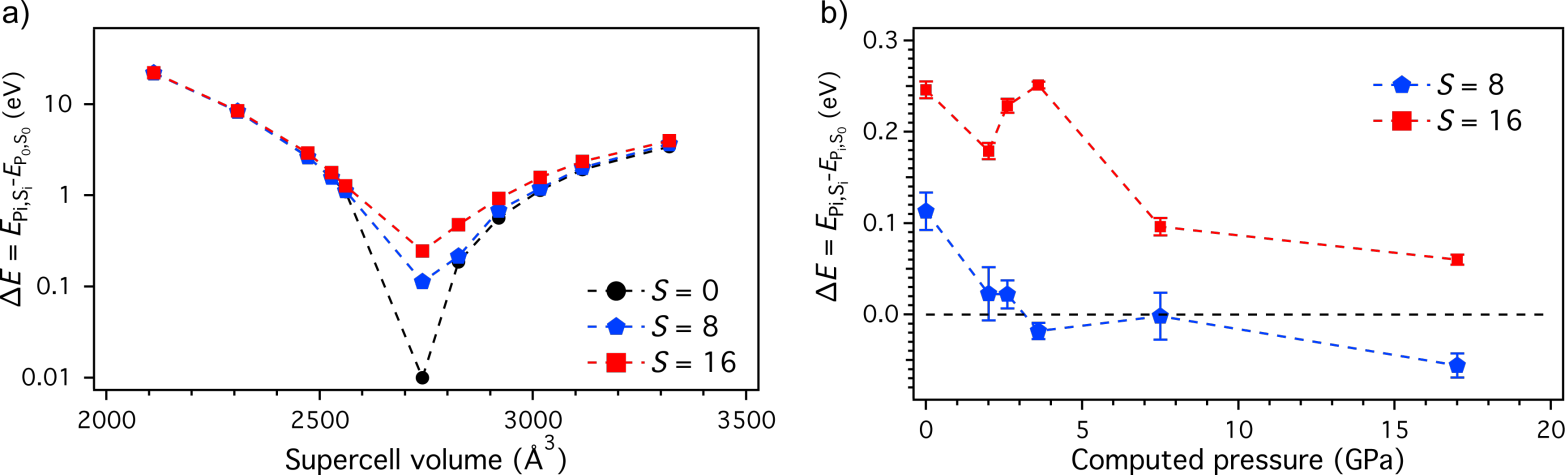
(a) Total energy of the simulated systems as a function of their volumes. We show the data for the three magnetization states: *S* = 0 in black circles, *S* = 8 in blue pentagons, and *S* = 16 in red squares. The data is normalized with respect to the energy of the ground state, found for the system with *S* = 0 at *P* = 0 GPa. (b) Relative stability of the systems with *S* = 8 and *S* = 16 shown in terms of total energy difference (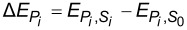) with respect to the *S* = 0 state (black dashed line) as a function of the applied pressure.

Figure 1a shows the total energy of the systems as a function of their volume for three different magnetization states with total spin multiplicities equal to 1 (*S* = 0), 17 (*S* = 8), and 33 (*S* = 16). In the absence of any external pressure, the *S* = 0 configuration corresponds to the ground state. In particular, this configuration is approx. 0.1 eV and approx. 0.25 eV more stable than the systems with *S* = 8 and *S* = 16, respectively. This energy difference between the states *S* = 0, *S* = 8, and *S* = 16 reduces significantly with increasing the pressure. The configuration with *S* = 8 becomes more stable than the one with *S* = 0 for pressures of ca. 3 GPa (Figure 1b), in fair agreement with the experimental findings (ca. 1.2 GPa) [4].

Notably, the AF-to-F transition pressure is closer to the experimental value than the one found in previous theoretical investigations (ca. 7.5 GPa) [7]. From a structural point of view, upon compression both Cu–O and Cu–Cu interatomic distances decrease and Cu–O–Cu angles are affected in order to adjust to the compression, in line with what reported in our previous theoretical works [7].

**Figure 2:**
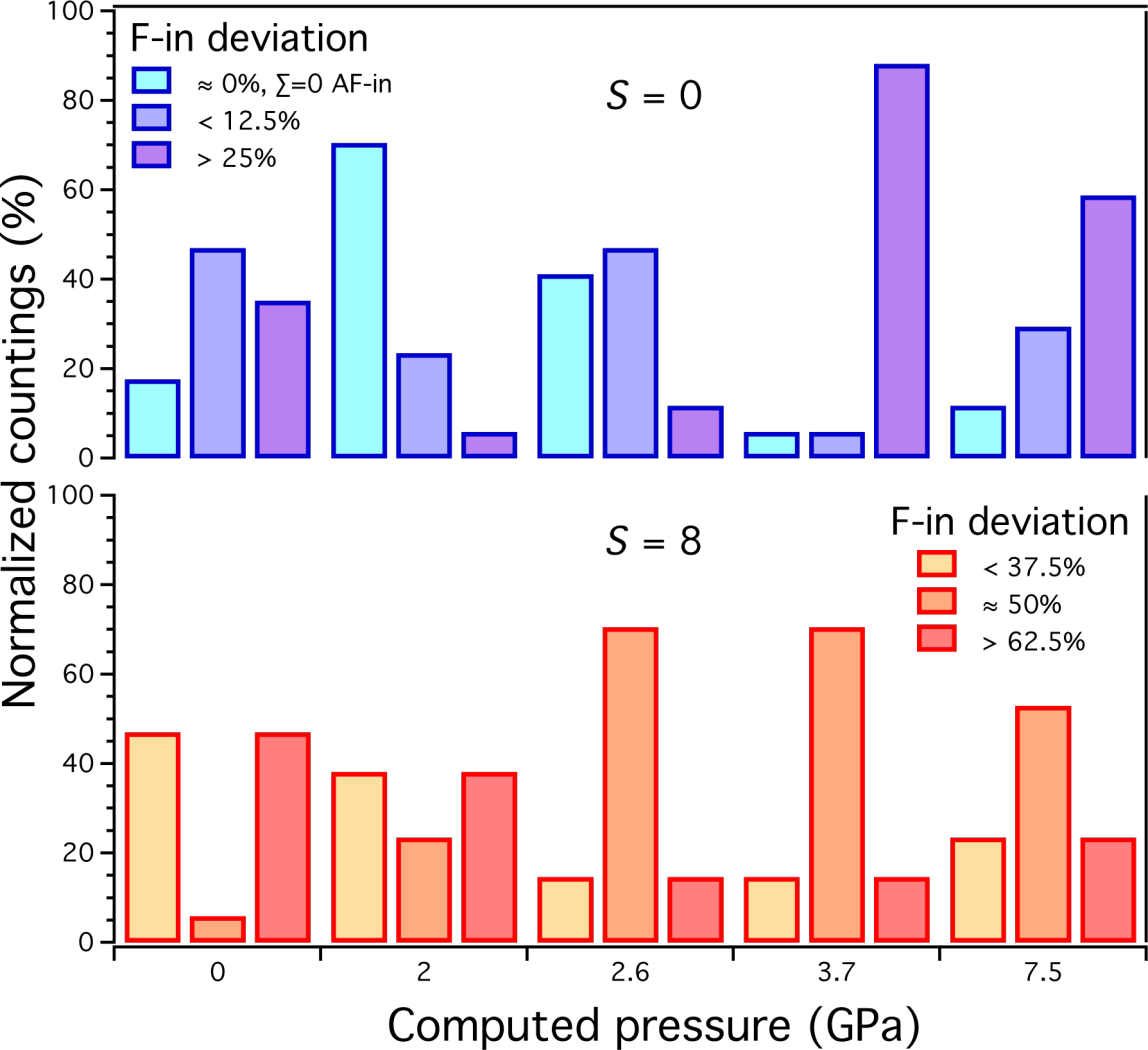
Distribution of the intralayer spin configurations for the systems with *S* = 0 (top) and *S* = 8 (bottom) as a function of pressure. For each pressure we split the spin distributions showing percentages of deviation with respect to the in-layer Σ = 0 case. For *S* = 0 we show deviations corresponding to one (i.e., 12.5%) and two or more (i.e., *>*25%) up(down)-spin of difference between the two layers. For *S* = 8 we show F-in corresponding to three or less (i.e., *<*37.5%), four (i.e., 50%), and five or more (i.e., *>*62.5%) up(down)-spin of difference between the two layers. Note that F-in deviation of 50% corresponds to an equal distribution between the two layers of the total spin for *S* = 8.

**Figure 3:**
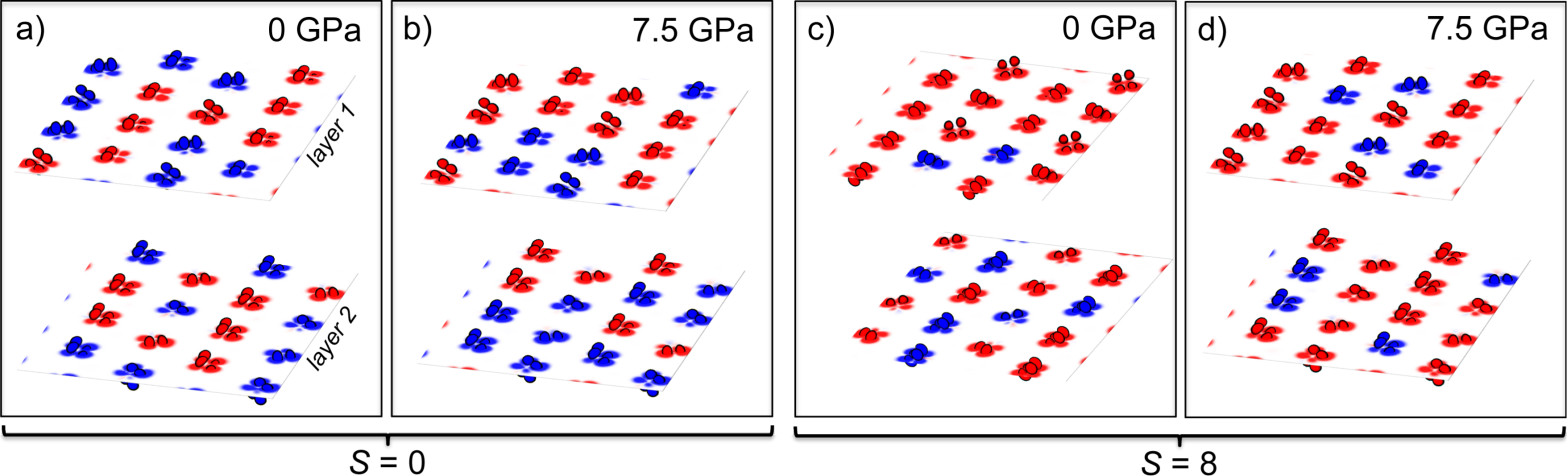
Volume-slice representations of the local spin configurations for the systems with *S* = 0 and *S* = 8 at pressure 0 GPa and 7.5 GPa. Volume slices centered along the two copper hydroxide planes per system, named layer 1 (L_1_) and layer 2 (L_2_). For each layer, the number of up- and down-spins are specified in the following: a) L_1_: 8 (up-spin)-8 (down-spin); L_2_: 8-8; b) L_1_: 10-6; L_2_: 6-10; c) L_1_: 2-14; L_2_: 6-10; d) L_1_: 4-12; L_2_: 4-12. These representations correspond to F-in deviations of ca. 0% (a) and *>*25% (b) for *S* = 0 and *>*62.5% (c) and ca. 50% (d) for *S* = 8, respectively.

We report in Figure 2 the 2D spin configuration as a function of the pressure. In particular, we analyze the spin distribution in terms of the intralayer deviation (F-in) from the case Σ = 0. To this purpose the spin distributions are shown by highlighting the different values taken with respect to this reference value (i.e., 0%) and the maximally spin polarized layer case (i.e., 100% F-in). Typical examples of the obtained spin distributions are shown in Figure 3 for *S* = 0 and *S* = 8 at 0 GPa and 7.5 GPa. By looking at the partial populations with different F-in deviation, the following tendencies can be observed. For *S* = 0, the pressure induces a decrease of the spin distributions with Σ = 0 whereas the content of spin distributions with a large 2D spin polarization (F-in *>* 25%) increases significantly. Conversely, for *S* = 8, an opposite trend can be noticed, with a decrease of the 2D spin polarized distributions (F-in *>* 62.5%) with pressure and, simultaneously, an increase of the populations with a lower polarization (F-in = 50%).

**Figure 4:**
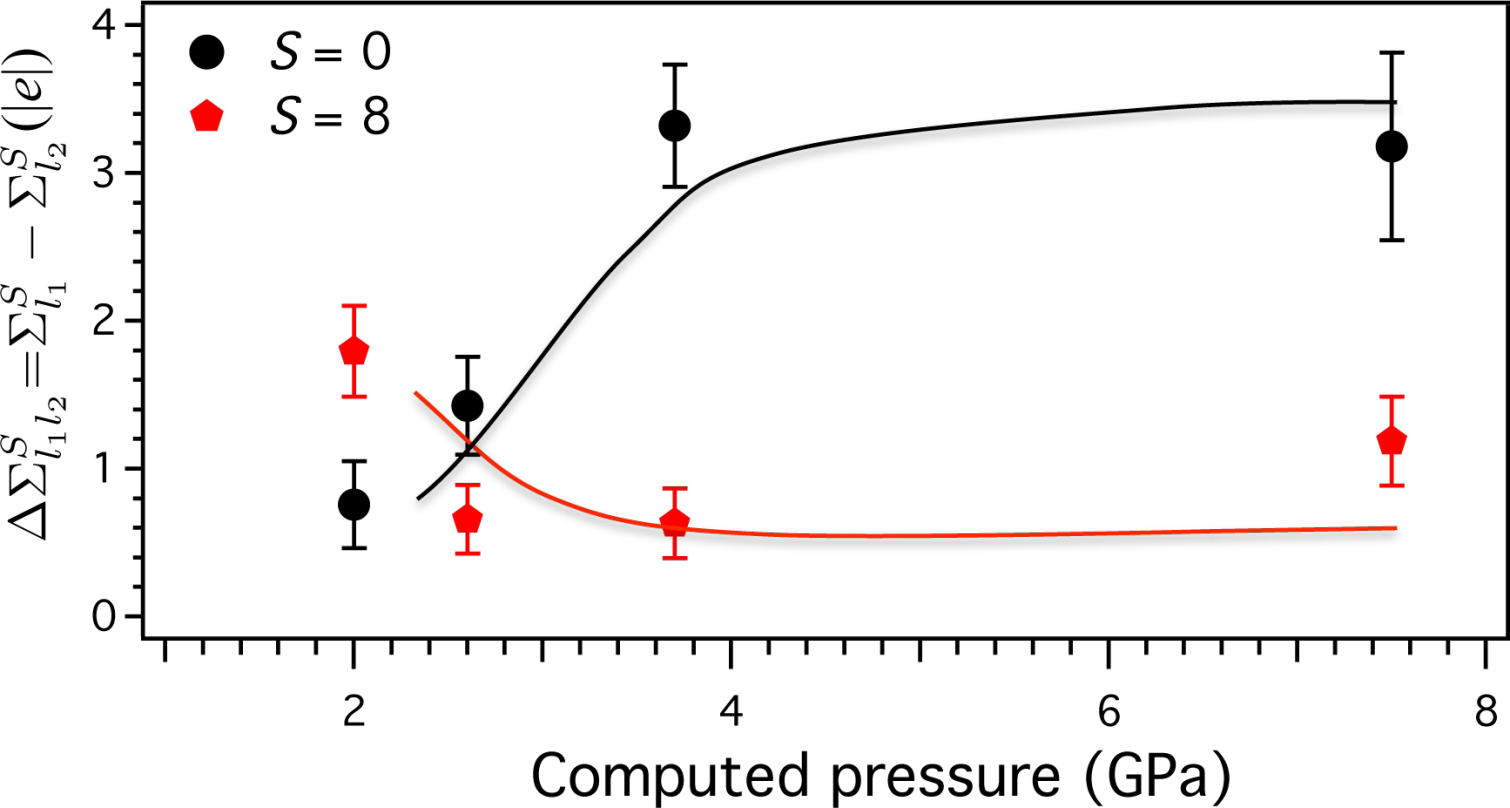
Difference of spin density between the two inorganic layers per system for *S* = 0 (black circles) and *S* = 8 (red pentagons) as a function of the pressure. Solid lines are guides to the eye.

Following the same scheme, by plotting the difference of interlayer spin 
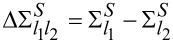
 we can estimate the degree of 3D spin polarization couplings of the system (Figure 4). For *S* = 0 the difference 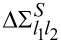 increases upon compression whereas for *S* = 8 the difference 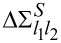 decreases. The cross-linking between these two trends, at about the AF-to-F transition pressure, suggests that the driving force to induce this transition is the tendency of the system to acquire equal values of finite spin polarization on each layer. Eventually, the most stable system under pressure features *S* = 8 as resulting from equal contributions of *S*_1_ = 4 and *S*_2_ = 4 on each layer. This spin configuration is the best suited to optimize the overall ferromagnetic character of this compound.

## Conclusion

We show that in Cu_2_(OH)_3_CH_3_COO·H_2_O the net effect of an external pressure is to affect both the structural properties as well as the intra- and inter-layer magnetic interactions of the inorganic layers. The novel set of results reported in the present study allows us to estimate a AF-to-F transition pressure upon compression in better agreement with the experimental findings than the previously reported. We found that the driving force underlying the magnetic transition is the trend toward a uniform spin polarization across the layers, ensuring an overall optimal ferromagnetic character.

## References

[1] Rabu P, Drillon M, Agawa K, Fujita W, Sekine T, Miller J S, Drillon M (2001). Hybrid Organic-Inorganic Multilayer Compounds: Towards Controllable and/or Switchable Magnets. Magnetism: Molecules to Materials II: Models and Experiments.

[2] Rogez G, Massobrio C, Rabu P, Drillon M (2011). Chem Soc Rev.

[3] Rabu P, Delahaye E, Rogez G (2015). Nanotechnol Rev.

[4] Suzuki K, Haines J, Rabu P, Inoue K, Drillon M (2008). J Phys Chem C.

[5] Yang F, Boero M, Massobrio C (2010). J Phys Chem C.

[6] Yang F, Boero M, Rabu P, Massobrio C (2012). C R Chim.

[7] Yang F, Massobrio C, Boero M (2014). J Phys Chem C.

[8] Banerjee H, Chakraborty S, Saha-Dasgupta T (2016). Chem Mater.

[9] Banerjee H, Kumar M, Saha-Dasgupta T (2014). Phys Rev B.

[10] Švarcová S, Klementová M, Bezdička P, Łasocha W, Dušek M, Hradil D (2011). Cryst Res Technol.

[11] Becke A D (1988). Phys Rev A.

[12] Lee C, Yang W, Parr R G (1988). Phys Rev B.

[13] Troullier N, Martins J L (1991). Phys Rev B.

[14] Louie S G, Froyen S, Cohen M L (1982). Phys Rev B.

